# Multimodality Treatment for Hepatocellular Carcinoma With Portal Vein Tumor Thrombus: A Large-Scale, Multicenter, Propensity Mathching Score Analysis

**DOI:** 10.1097/MD.0000000000003015

**Published:** 2016-03-18

**Authors:** Kang Wang, Wei Xing Guo, Min Shan Chen, Yi Lei Mao, Bei Cheng Sun, Jie Shi, Yao Jun Zhang, Yan Meng, Ye Fa Yang, Wen Ming Cong, Meng Chao Wu, Wan Yee Lau, Shu Qun Cheng

**Affiliations:** From the Department of Hepatic Surgery (KW, WXG, JS, MCW, WYL, SQC), Eastern Hepatobiliary Surgery Hospital, The Second Military Medical University, Shanghai; Faculty of Medicine (WYL), The Chinese University of Hong Kong, Shatin, Hong Kong SAR; Department of Hepatobiliary Surgery (MSC, YJZ), SunYat-sen University Cancer Center; Department of Liver Surgery (YLM), Peking Union Medical College (PUMC) Hospital, Chinese Academy of Medical Sciences and PUMC, Beijing; Liver Transplantation Center of the First Affiliated Hospital (BCS), Nanjing Medical University, Nanjing, Jiangsu Province; Department of Radiotherapy (YM); Department of Invasive Technology (YFY); and Department of Pathology (WMC), Eastern Hepatobiliary Surgery Hospital, The Second Military Medical University, Shanghai, China.

## Abstract

The optimal treatment for hepatocellular carcinoma (HCC) with portal vein tumor thrombus (PVTT) remains controversial. We aimed to investigate the best treatment for patients with HCC with PVTT.

From January 2002 to January 2014, the data from all consecutive patients with HCC with PVTT who underwent surgical treatment (ST),TACE,TACE combined with sorafenib (TACE-Sor), or TACE combined with radiotherapy (TACE-RT) in the 4 largest tertiary hospitals in China were analyzed retrospectively. The patients were divided into 3 subtypes according to the extent of PVTT in the portal vein (type I-III). The primary endpoint was overall survival (OS).

A total of 1580 patients with HCC with PVTT were included in the study. The median survival times (MST) for ST (n = 745) for type I, II, and III patients (95% CI) were 15.9 (13.3–18.5), 12.5 (10.7–14.3), and 6.0 (4.3–7.7) months, respectively. The corresponding figures for patients after TACE (n = 604) were 9.3 (5.6–12.9), 4.9 (4.1–5.7), and 4.0 (3.1–4.9), respectively; for patients after TACE-Sor (n = 113) 12.0 (6.6–17.4), 8.9 (6.7–11.1), and 7.0 (3.0–10.9), respectively; and for patients after TACE-RT (n = 118) 12.2 (0–24.7), 10.6 (6.8–14.5), and 8.9 (5.2–12.6), respectively. Comparison among the different treatments for the 3 subtypes of PVTT patients after propensity score (PS) matching showed the effectiveness of ST to be the best for type I and type II PVTT patients, and TACE-RT was most beneficial for type III patients. Treatment was an independent risk factor of OS.

ST was the best treatment for type I and II PVTT patients with Child-Pugh A and selected B liver function. TACE-RT should be given to type III PVTT patients.

## Abstract 2

Supplemental Digital Content is available in the text

## INTRODUCTION

Hepatocellular carcinoma (HCC) is a common cancer with a dismal prognosis. Among factors which contribute to poor outcomes, portal vein tumor thrombus (PVTT) is most important.^1–3^ PVTT occurs in 12.5% to 39.7% of patients with HCC and up to 64.7% of HCC patients at autopsy. If left untreated, a median survival time (MST) of 2.7 to 4.0 months has been reported.^4,5^ Unfortunately, the optimal treatment for HCC with PVTT remains controversial.

The current treatment strategy for patients with HCC with PVTT differs in the West and in the East. The EASL guideline, which is commonly used in the West, recommends sorafenib to be the only treatment. On the other hand, the Asia-Pacific guideline recommends surgery, transhepatic arterial chemoembolization (TACE), radiotherapy (RT),^2,6^ and sorafenib as treatment options. Reports coming from the Asia-Pacific region showed the overall survival in patients with HCC with PVTT differs significantly with the type of treatment^7^ and with the extent of PVTT. There are currently 2 commonly used systems to classify the extent of HCC with PVTT: Cheng's Classification for PVTT (Type I–IV) and the Japanese staging system (Vp1-Vp4).^8–10^ The prognosis of patients and the treatment strategy for each subtypes of PVTT differ. Little is known about the impact on overall survival using different treatment strategies for the different subtypes of PVTT patients. Thus, little is known on how to select the most appropriate treatment for patients with HCC with a particular subtype of PVTT.

In this study, we analyzed the characteristics of Chinese HCC patients with PVTT and compared the effectiveness of ST, TACE, TACE combined with sorafenib (TACE-Sor), and TACE combined with RT (TACE-RT) for each subtype of PVTT based on Cheng's Classification. After propensity score matching, the long-term survival outcomes were analyzed.

## MATERIALS AND METHODS

### Diagnostic Criteria for PVTT

PVTT was diagnosed using radiologic imagings (CT, MRI, and/or ultrasound) and/or histopathology.^11^ Patients with macroscopic hepatic vein tumor thrombus (m-HVTT) were excluded from this study. Based on Cheng's Classification, PVTT was classified into 4 grades according to the extent of PVTT in the portal vein: Type I, tumor thrombus in the segmental branches of the portal vein or above; Type II, tumor thrombus extending to the right or the left portal vein; Type III, tumor thrombus extending to the main portal vein; and Type IV, tumor thrombus extending to the main portal vein and the superior mesenteric vein. The liver function and/or remnant liver volume were assessed using blood tests and CT volumetric studies.

### Patients and Design of the Study

We reviewed the demographic, clinical, and pathological data of consecutive patients with HCC with PVTT who underwent ST, TACE, TACE-Sor, or TACE-RT from January2002 to January 2014 in 4 centers in China (the participating organizations are shown in the acknowledgement). All centers involved in this study used the same standard laboratory methods for measurement of biochemical parameters. All patients who were included into this study (n = 1580) were divided into 3 subgroups according to Cheng's Classification for PVTT (Type I–III). Notably, there were insufficient data for the type IV PVTT patients (Supplement Table 1), and the prognoses of the different treatments for this group of patients were not analyzed. The data for the type I PVTT patients who received TACE combined with RT was also insufficiently small (only 8 patients. Supplement Table 2), and the results of this combined treatment were not compared with other treatments.

The treatments for type I PVTT patients included ST, TACE, and TACE-Sor. In addition, the treatments for type II-III PVTT patients also included TACE-RT. The prognosis of patients who underwent the different treatments in each subgroups was analyzed before and after propensity score matching.

### Inclusion Criteria

The inclusion criteria were: (1) Child–Pugh class A or selected B liver function; (2) patients with HCC with macroscopic PVTT diagnosed by radiologic imagings and/or histopathology; (3) no macroscopic hepatic vein tumor thrombus (m-HVTT); (4) no extrahepatic spread or distant metastases; (5) no other malignancies; (6) no concomitant use of other targeting agents, chemotherapy, or immunotherapy; (7) HCV-related HCC or HCC with mixed etiologies were excluded.

The study protocol was approved by the Institutional Ethics Committee (IEC) of the participating hospitals. Written informed consent was obtained from all the patients for their data to be used for research.

### Surgery Procedures

The surgical procedures have been reported in our previous study.^12^ Only patients with Child–Pugh A or selected B liver function were offered hepatic resection. Patients with type IV PVTT were not considered for surgery.

During surgery, routine intraoperative ultrasonography was carried out for assessment of resectability by detection for major vascular invasion in the contralateral lobe and undetected tumors in the future liver remnant. We carefully searched the abdominal cavity for extent of local disease, extrahepatic metastases, and peritoneal seeding. The blood inflow of the liver was occluded using Pringle's maneuver. The clamp crushing method was used to carry out liver resection.

Thrombectomy was performed according to the types of PVTT. For patients with Type I, IIPVTT, the PVTT was resected en bloc with the specimen. For patients with Type IIIPVTT with the PVTT protruding into the main portal vein beyond the resection line, the main portal trunk was dissected, controlled with vascular clamps to the distal PVTT, and opened. The PVTT was extracted. The lumen was flushed with normal saline to remove potentially cancerous residual tissue. The stump was closed by a continuous suture.

### TACE Procedures

TACE was performed in patients who were not eligible or unwilling to receive ST. After TACE, some patients elected to have TACE-Sor, whereas others combined with RT. Seldinger's technique was used. Contrast medium was injected *via* a selective 5-F RH catheter (Cook, Bloomington, Ind) or Cobra catheter (Cook) or microcatheter (Renegade, Boston Scientific, Natick, Mass; Progreat, Terumo, Tokyo, Japan) through the sectoral, segmental, or subsegmental hepatic arteries, based on the size, location, arterial supply of the tumor and hepatic functional reserve. An emulsion of 20 to 60 mg doxorubicin hydrochloride, cisplatin (5 mg), and lipiodol (LipiodolUltrafluide, Guerbet, Aulnay-Sous-Bois, France) 5 to 30 mL (1 to 2 mL/cm diameter of the tumor) were injected through the catheter; Gelfoam fragments were then injected to embolize the tumor-feeding vessel. The dosages of lipiodol and doxorubicin were determined by tumor size, vascularity, presence of arterio portal shunt, and underlying liver function. After 1 month, follow-up computed tomography (CT) was performed. Based on liver function and tumor response, TACE was repeated at intervals of 6 to 8 weeks if intrahepatic residual viable tumor was found. Repeat TACE treatment was halted if the patient's liver function deteriorated until the liver function had recovered (Child-Pugh A-B). TACE was stopped when the tumors failed to respond and progressed with treatment.

### TACE Combined With Sorafenib

Sorafenib was administered orally at a dosage of 400 mg twice daily at 1 week after the first TACE session. It was continued with no dose reduction before or after repeated TACE unless toxicity as defined by the National Cancer Institute Common Terminology Criteria for Adverse Events (or NCICTCAE) developed.^13^ For grade 3 or 4 adverse events, the dosage was reduced to 200 mg twice daily until the adverse events were alleviated or eliminated. For more significant toxicity the drug was discontinued until the adverse effects were alleviated or disappeared.

### TACE Combined With Radiotherapy

TACE was first carried out. After an interval time of 2 to 4 weeks, RT was then carried out for the portal tumor thrombus which was outlined as the clinical target volume (CTV). On follow-up, if intrahepatic residual viable tumor or recurrent tumor was found on CT/MRI, TACE was repeated.

The RT procedure: after fixing by the vacuum pad or the phantom, the patient was scanned with a slice thickness of 5.0 mm on arterial phase and portal phase, from the carina to the fifth lumbar vertebra. The image data and the related data were delivered to the treatment plan system (TPS). The portal tumor thrombus was outlined as CTV, and the plan target volume (PTV) was expanded 1.0 cm in the direction of the XY axis, 0.5 cm in the Z axis (head direction). Prescribed doses to the initial PTV ranged from 50 to 66 Gy (median 56 Gy) in daily doses of 2.0 to 2.2 Gy. Biologically effective dose (BED) ranged from 60 Gy to 80.5 Gy (median 67.2 Gy, α/β = 10). Dose–volume histogram (DVH) was used for dose optimization, with 90% dose curve completely covered by the PTV. The internal dose of tumor was uniform, and the dose change was not >5%. The dose to other organs such as the gastrointestinal tract and the spinal cord were all acceptably low, and the dose limit of a high dose was not >10%.

### Follow-Up and Survival Analyses

For patients who received ST, the patients were followed-up once every 3 to 4 months until death or dropout from the follow-up program. When recurrent HCC was diagnosed, the patients were actively treated with radiofrequency ablation, percutaneous ethanol injection, transarterial chemoembolization, or repeat liver resection, based on the general condition of the patient, the underlying liver functional status, and the number and location of HCC recurrence. A diagnosis of recurrence of HCC was based on computed tomography and/or magnetic resonance imaging and raised serum a-fetoprotein (AFP) level. For patients who received TACE, TACE-Sor, and TACE-RT, they were followed up once every 6 to 8 weeks to decide whether they required another session of TACE. Overall survival (OS) was defined as the interval (in months) from the date of receiving the first treatment to the date of death or the last follow-up, and it was used to evaluate the effectiveness of each treatment.

### Statistical Analysis

A propensity score (PS) matching model was used for accurate comparison between the modalities. The PS procedure was according to the “Propensity score matching in SPSS” of Cornell University Library.^14^ Variables potentially affecting the outcomes were assigned propensity score after logistic regression analysis. The nearest neighbors in each group were matched 1-to-1 or 1-to-3 based on the generated propensity scores using a caliper width of 0.15 and no replacement.^15^

After PS matching, survival was analyzed by the Kaplan–Meier method and survival curves were compared by the log-rank test. Univariable and multivariable analyses were based on the Cox proportional analysis, the treatment strategies and type of PVTT were plugged into Cox proportional analysis as categorical covariates. Categorical variables were compared by the chi-square test or Fisher exact test. A value of *P* < 0.05 was statistically significant. The analysis was performed with the SPSS for Windows 22 and R for Windows 2.15.3.

## RESULT

### The Characteristics Data Before and After PS Matching

From 2002 to 2014, 1580 patients with HCC with PVTT coming from 4 centers in China were enrolled into this study, 745 underwent ST, 604TACE, 113 TACE-Sor, and 118 TACE-RT. There were significant differences in the baseline characteristics of the 4 treatments in each of the subtypes of PVTT before PS matching (Table 1 ). Patients who underwent ST had better liver function and less tumor number than the counterparts in each of the other subtypes of PVTT.

**TABLE 1:**
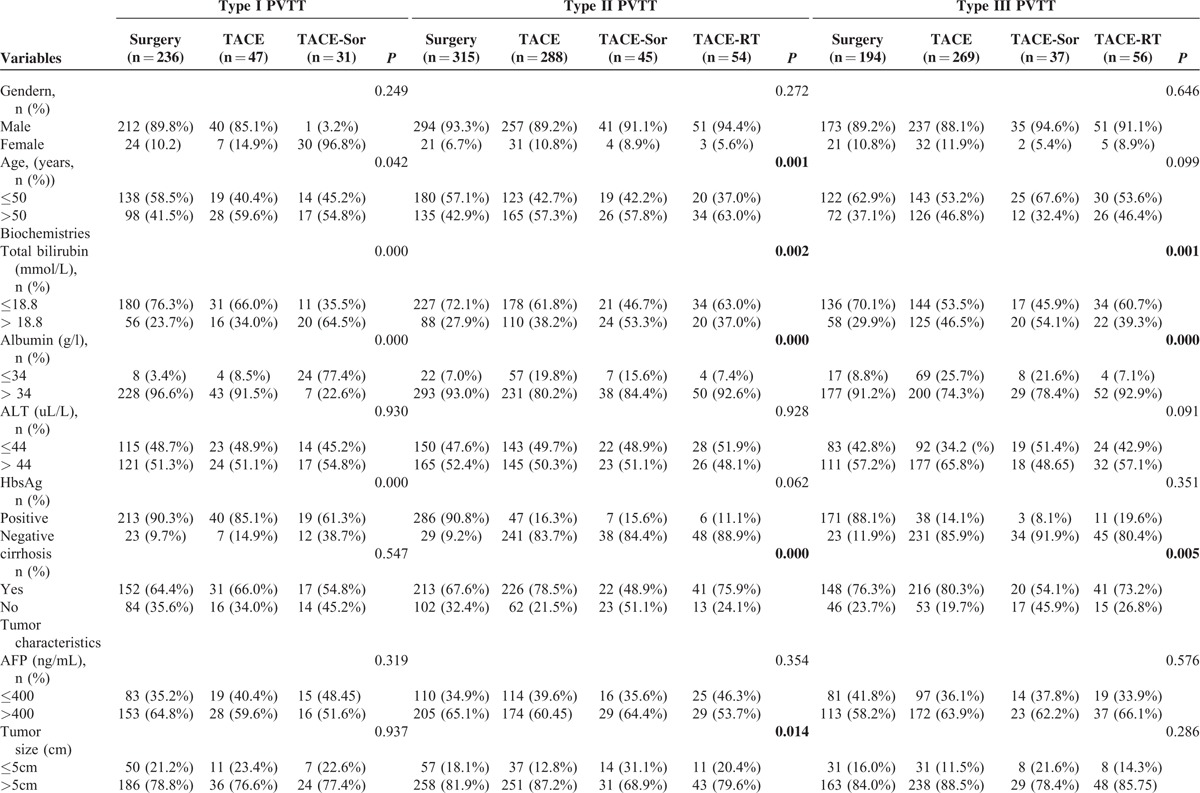
Patient's Characteristics of All Enrolled Patients

**TABLE 1 (Continued):**
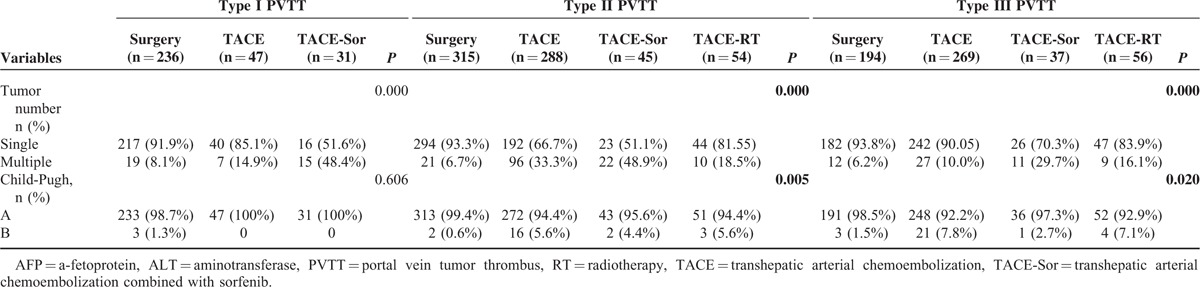
Patient's Characteristics of All Enrolled Patients

In addition (Table 1 ), there were significant differences in age and HbsAg positivity for type I PVTT patients between the multiple treatment groups (*P* = 0.042 and *P* < 0.001 in age and HbsAg positivity, respectively). For type II PVTT patients who underwent ST had significantly smaller tumor size and less severe cirrhosis than their counterparts (*P* = 0.014 and *P* < 0.001 for tumor size and liver cirrhosis, respectively). Age was also significantly different for this group of patients (*P* = 0.001). For type III PVTT patients, the severity of liver cirrhosis was significantly different among ST and the counterparts (*P* = 0.005).

Supplement Tables 3 to 8, show the comparison between the groups of patients who underwent multiple treatments for each of the different types of PVTT. As expected, there were significant differences in the baseline characteristics. After PS matching, these characteristics became well balanced. Notably, no PS matching was carried out for the characteristics of type III PVTT patients who underwent TACE-Sor or TACE-RT because the raw data were well balanced (Supplement Table 7).

### Survival Analysis

Before PS matching, the survival profiles of ST, TACE, TACE-Sor, and TACE-RT for type I to III PVTT patients are shown in Table 2 and Supplement Figure 1B–D. For type I and type II PVTT patients, the MST (95% CI) of patients after ST was significantly longer than their counterparts (15.91 (13.278–18.542) and was 12.51 (10.718–14.302) for type I and type II PVTT patients, respectively, *P* < 0.001). For type III PVTT patients, the survival after TACE was the worst (3.98 [3.088–4.872], *P* = 0.001). The MSTs (95% CI) among ST, TACE-Sor, and TACE-RT were not significantly different (6.01 [4.346–7.674], 6.96 [3.015–10.905], and was 8.9 [5.197–12.603] for ST, TACE-Sor, and TACE-RT, respectively, *P* = 0.063).

**TABLE 2:**
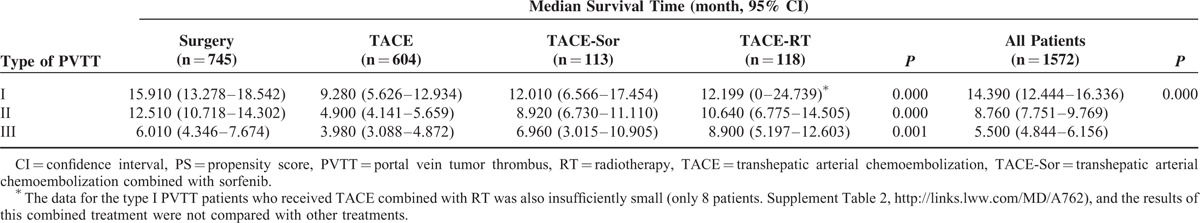
The Median Survival Time of the Enrolled Patients Before PS Matching

Before PS matching, the survival profiles of the subtypes of PVTT patients are shown in Table 2 and Supplement Figure 1A. The MSTs (95% CI) were14.39 (12.444–16.336), 8.76 (7.751–9.769), and 5.5 (4.844–6.156) for type I, type II, and type III PVTT patients, respectively, *P* < 0.001.

**FIGURE 1:**
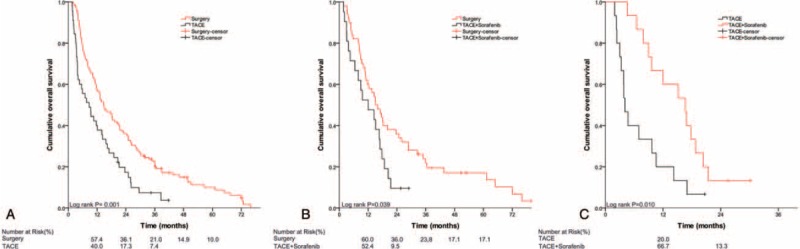
Kaplan–Meier analysis for overall survival (OS) in patients with HCC with type I PVTT who underwent different treatments: (A) surgery versus TACE; (B) surgery versus TACE combined with sorafenib; (C) TACE versus TACE combined with sorafenib. HCC = hepatocellular carcinoma, OS = overall survival, PVTT = portal vein tumor thrombus, TACE = transcatheter arterial chemoembolization.

**FIGURE 2:**
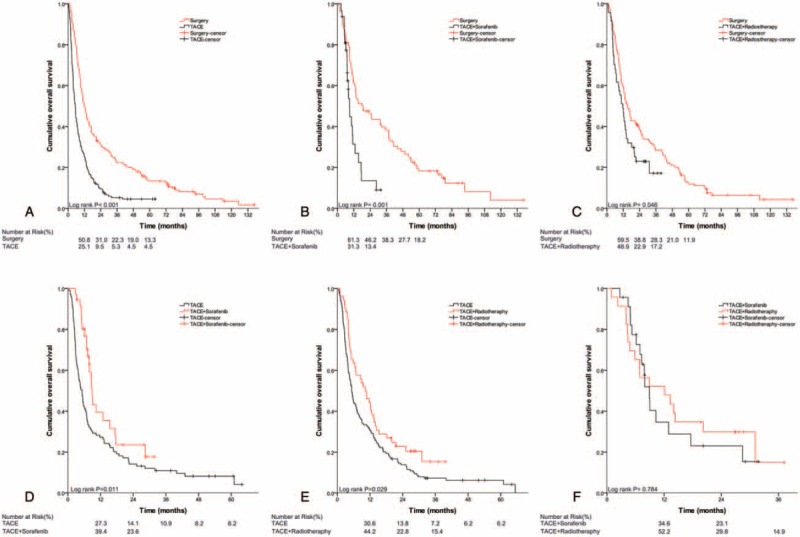
Kaplan–Meier analysis for overall survival (OS) in patients with HCC with type II PVTT who underwent different treatments: (A) surgery versus TACE; (B) surgery versus TACE combined with sorafenib; (C) surgery versus TACE combined with radiotherapy; (D) TACE versus TACE combined with sorafenib; (E) TACE versus TACE combined with radiotherapy; (F) TACE combined with sorafenib versus TACE combined with radiotherapy. HCC = hepatocellular carcinoma, OS = overall survival, PVTT = portal vein tumor thrombus, TACE = transcatheter arterial chemoembolization.

**FIGURE 3:**
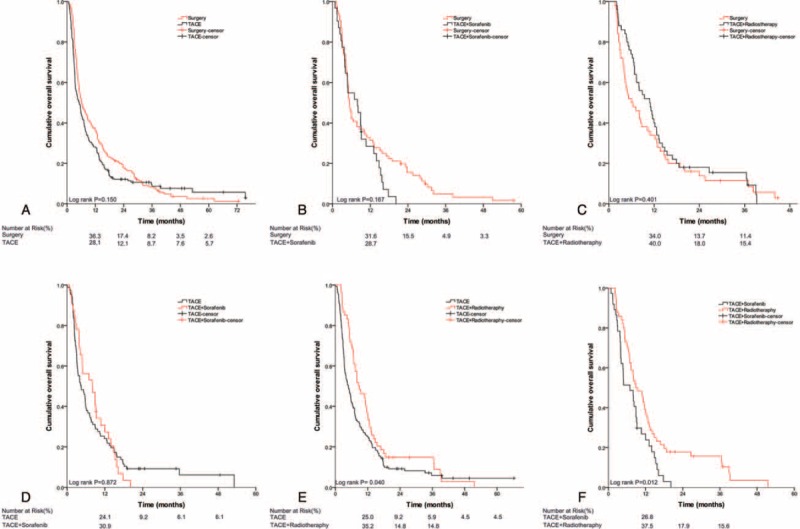
Kaplan–Meier analysis for overall survival (OS) in patients with HCC with type III PVTT who underwent different treatments: (A) surgery versus TACE; (B) surgery versus TACE combined with sorafenib; (C) surgery versus TACE combined with radiotherapy; (D) TACE versus TACE combined with sorafenib; (E) TACE versus TACE combined with radiotherapy; (F) TACE combined with sorafenib versus TACE combined with radiotherapy. HCC = hepatocellular carcinoma, OS = overall survival, PVTT = portal vein tumor thrombus, TACE = transcatheter arterial chemoembolization.

The comparisons among multiple treatments for subtypes of PVTT patients after PS matching are shown in Table 3 and Figure 1, Figure 2, and Figure 3. The results showed ST to result in better OS than the counterparts (Figure 1A and B, Figure 2A–C) for type I and II PVTT patients, the corresponding MSTs (95% CI) are shown in Table 3. For type III PVTT patients, TACE-RT produced better OS than TACE or TACE-Sor (Figure 3E and F).

**TABLE 3:**
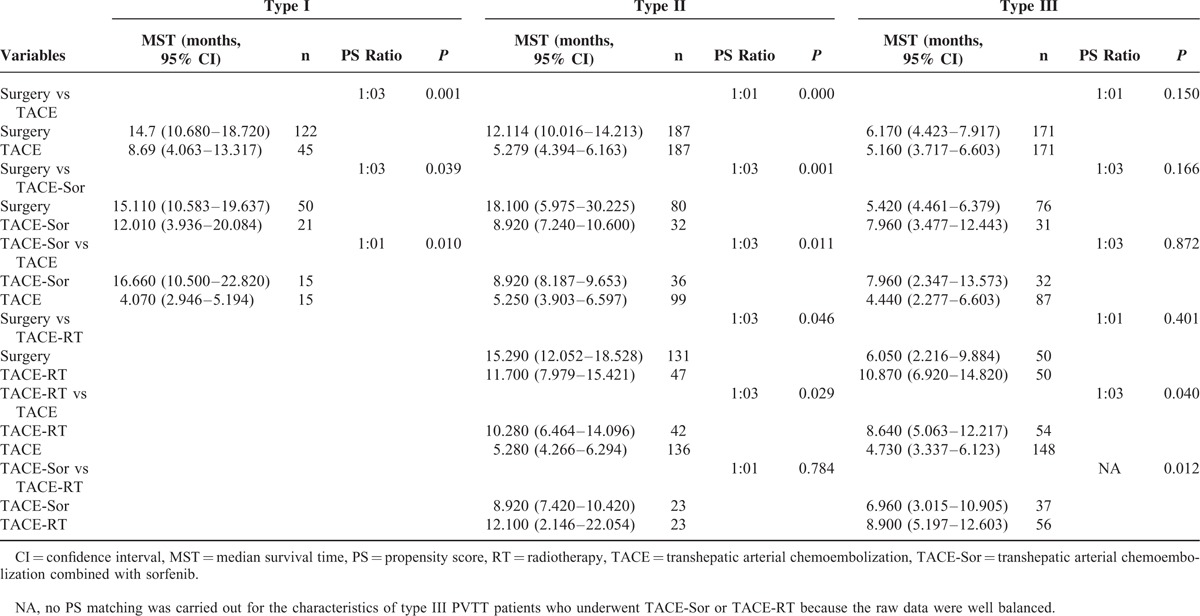
The Median Survival Time of the Enrolled Patients After PS Matching

On Cox's multivariable analysis for all the enrolled patients (n = 1580), the treatment and the type of PVTT were independent risk factor of OS (Table 4). The corresponding hazard ratios (HR) (95% CI) were: 1.784 (1.543–1.979), *P* < 0.001 for TACE versus (vs) surgery + TACE-Sor + TACE-RT; 1.340 (1.065–1.687), *P* = 0.013 for TACE-Sor versus TACE + surgery + TACE-RT; 1.095 (0.876–1.370), *P* = 0.425 for TACE-RT versus TACE + surgery + TACE-Sor. The MSTs for type I versus type II + III and type II versus type I+III PVTT patients were 0.682 (0.583–0.797), *P* < 0.001 and/or 0.703 (0.622–0.795), *P* < 0.001, respectively.

**TABLE 4:**
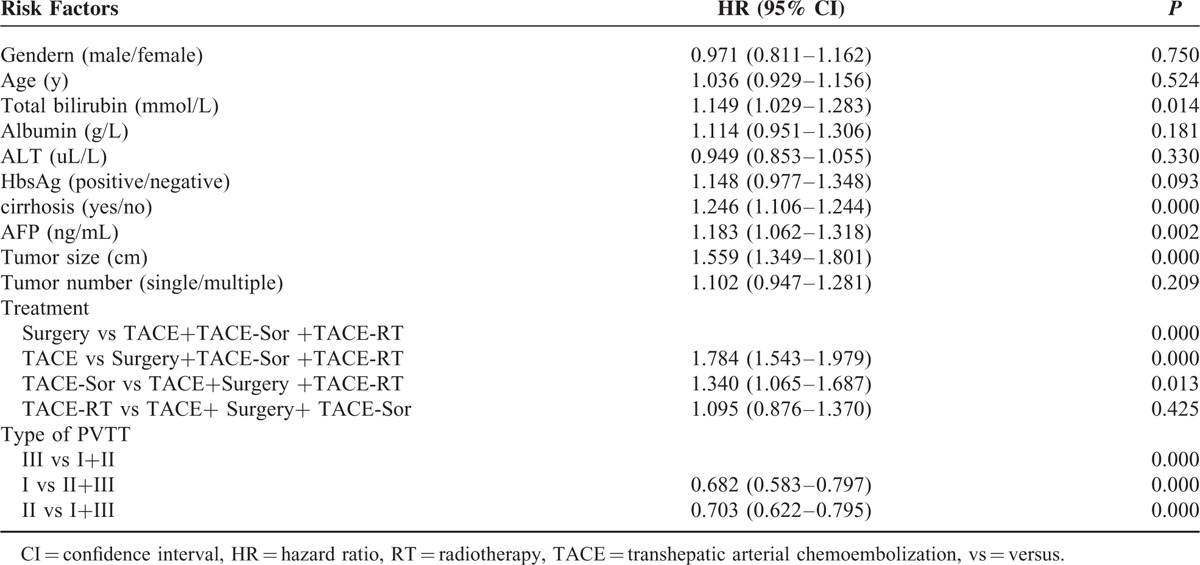
Multivariate Analysis to Identify Prognostic Factors Associated With Survival in All Enrolled Patients

## DISCUSSION

PVTT is one of the most unfavorable prognostic factors of overall survival in patients with HCC. Although a number of therapeutic modalities have been proposed, there is no worldwide consensus on the management of such patients. To the best of our knowledge, our study first compared the overall survival of patients who underwent different treatments in a multicenter, large cohort of patients with HCC with PVTT. Our results indicated that ST is the best treatment for type I and II PVTT patients with Child-Pugh A and selected B liver function. TACE combined with RT could be attempted in type I and II PVTT patients who are not candidates for surgery, and in selected type III PVTT patients.

ST for patients with HCC with PVTT has been advocated by various authors,^16,17^ producing a MST of 6.2 to 64.0 months, an operative mortality of 0 to 5.9 % and 1-, 3-, and 5-year survival rates in type I-III PVTT of 16% to 85%, 5.7% to 68%, and 0% to 61%, respectively.^7^ In our previous study, we suggested that ST was justified in type I and type II PVTT patients.^12^ Our present study further suggested that type III PVTT is not an absolute contraindication to ST in selected patients. For type I and II PVTT patients, ST significantly prolonged the patients’ survival when compared to other treatments. There is little doubt that a selection bias existed in this study and better patients were selected to undergo ST. In the unmatched data (Table 1 ), patients who underwent ST had significantly better liver function and less tumor number. However, after PS matching, significantly better OS was found for type I and II PVTT patients with Child-Pugh A or selected B liver function, and with good general conditions and resectable tumors. R0 resection should be attempted in these patients. For type III PVTT patients, our result indicated ST was not superior to other treatments. In this case, the choice of treatment should be fully considered against the background of the patients’ general conditions, liver function, the characteristics of tumor, and the patients’ wish. For type IV PVTT patients they are not suitable for surgical resection and they have poor prognoses.

TACE for PVTT patients has gradually gained acceptance, especially after the 2010 International Hepato-Pancreato-Biliary Association expert consensus statement.^18^ MSTs of 7 to 32 months have been reported.^19,20^ In our study, the MSTs for type I, II, and III PVTT patients were 9.28, 4.9, and 4.0 months, respectively (Table 2). In our study TACE did not produce better overall survival than TACE-Sor or TACE-RT. The reported MST of patients with HCC with PVTT treated with sorafenib is short, only 6.5 months in the Asian-Pacific region^21^ (10.5 months for the western population^22^).TACE-Sor for PVTT patients has been reported to be better than TACE in a small sample study.^23^ However, a propensity score matching study has failed to come to this result.^24^ Thus, this treatment was controversial and maybe given to selected patients with HCC with PVTT.

In recent years, with gradual improvement in RT technology, RT has become an effective method to treat HCC with PVTT. The reported MSTs after RT is 6.3 to 27.6 months.^25,26^ RT has the obvious superiority of protecting liver function and improving tumor control rate. According to reported studies for patients with HCC with PVTT, RT can result in a better curative effect, with mild adverse reaction. It is usually well-tolerated by patients and it prolongs survival^27–29^_ENREF_29. A present study indicated that TACE combined with RT is a hopeful strategy for unresectable type II PVTT patients, especially for type III PVTT patients. The results of the combined treatment were superior to ST, TACE, and TACE-Sor. In our study, we were unable to compare TACE combined with RT to other treatments due to insufficient data for the type I PVTT patients. The MST for the 8 patients with type I PVTT who received TACE combined with RT was 12.2 (0–24.7) months, which was shorter than ST. There was probably a selection bias in this small sample size. We are now conducting a clinical study to validate the results comparing ST with RT combined with TACE.

The limitations of this study are: first, the study was performed in China which has a high proportion of HBV infection. Second, the retrospective nature of the study is vulnerable to potential biases. Even with PS matching, these biases may still exist.

In conclusion, our results indicated that ST produced better overall survival for type I and II PVTT patients with Child-Pugh A and selected B liver function and resectable tumors. For type III patients, TACE combined with RT should be attempted. TACE-Sor could be used in type I to III PVTT patients who are not candidates for ST or RT.

## Supplementary Material

Supplemental Digital Content
