# Supplementary material for: Multimodality Treatment for Hepatocellular Carcinoma With Portal Vein Tumor Thrombus: A Large-Scale, Multicenter, Propensity Mathching Score Analysis
Source: Medicine (Baltimore). 2016 Mar 18;95(11):e3015. doi: 10.1097/MD.0000000000003015 (PMC4839896; doi:10.1097/MD.0000000000003015)
Supplement: Supplemental Digital Content [file medi-95-e3015-s001.doc]

**
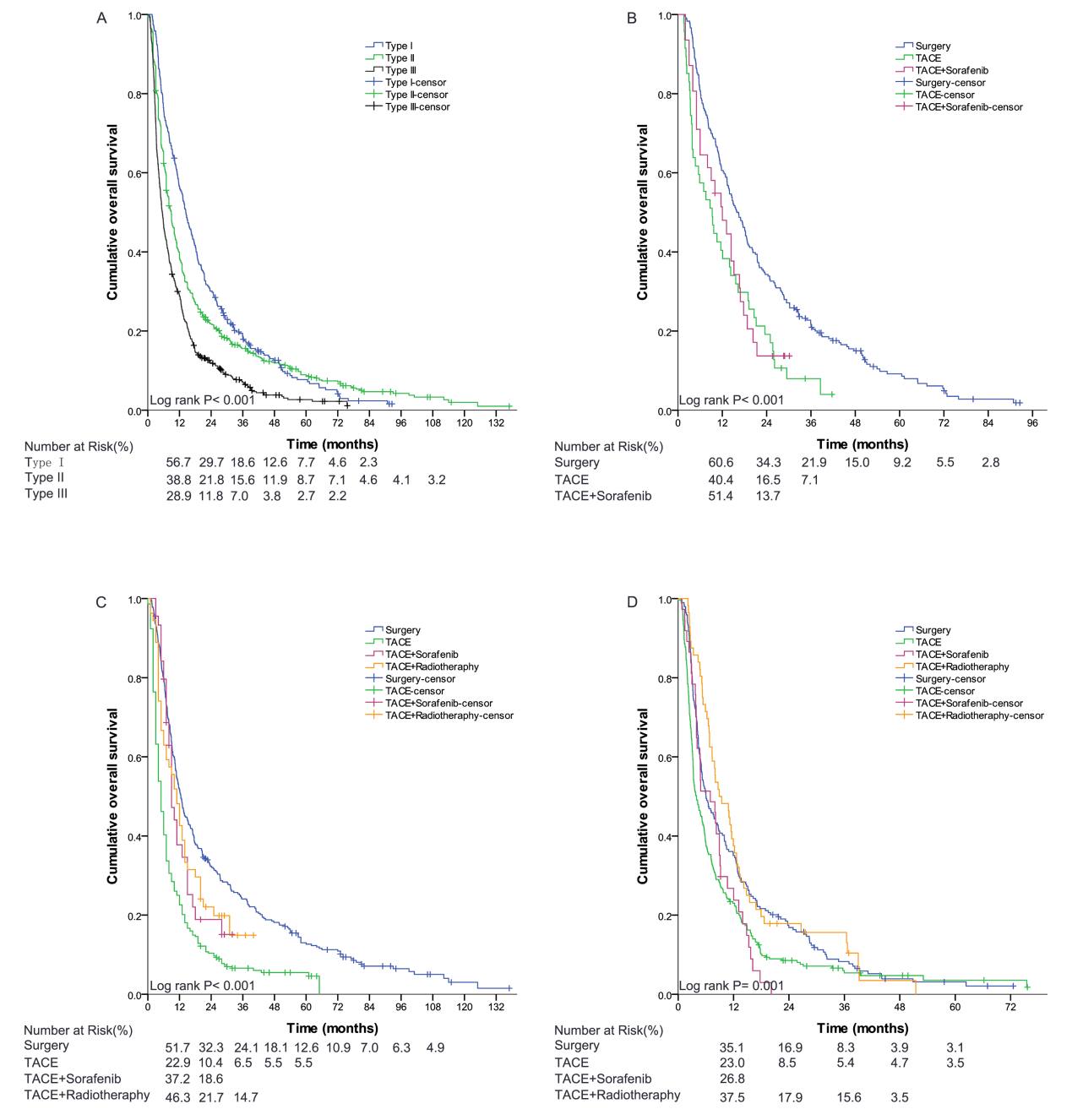
**

**Supplement Figure 1**. Kaplan-Meier analysis for overall survival (OS) in patients with HCC with PVTT

A) The OS for the subtypes of PVTT;

B) The OS for type I PVTT patients who underwent different treatments;

C)The OS for type II PVTT patients who underwent different treatments;

D)The OS for type III PVTT patients who underwent different treatments.

**Supplement Table 1 Patient’s Characteristics and Survival for Type IV PVTT patients**

|  | TACE | TACE-Sor |  |
| --- | --- | --- | --- |
| Variables | (n=13) | (n=7) | P |
| Gendern , n(%) |  |  | 0.452 |
| Male | 12(92.3%) | 7(100%) |  |
| Female | 1(7.7%) | 0 |  |
| Age, (years, n(%)) |  |  | 0.101 |
| ≤50 | 9(69.2%) | 7(100%) |  |
| >50 | 4(30.8%) | 0 |  |
| Biochemistries |  |  |  |
| Total bilirubin(mmol/L), n(%) |  |  | 0.081 |
| ≤18.8 | 9(69.2%) | 2(28.6%) |  |
| > 18.8 | 4(30.8%) | 5(71.4%) |  |
| Albumin (g/l), n(%) |  |  | 0.658 |
| ≤34 | 5(38.5%) | 2(28.6%) |  |
| > 34 | 8(61.5%) | 5(71.4%) |  |
| ALT (u l/1), n(%) |  |  | 0.423 |
| ≤44 | 8(61.5%) | 3(42.9%) |  |
| > 44 | 5(38.5%) | 4(57.1%) |  |
| HbsAg n (%) |  |  | 0.948 |
| Positive | 2(15.4%) | 1(14.3%) |  |
| Negative | 11(84.6%) | 6(85.7%) |  |
| cirrhosis n (%) |  |  | 0.357 |
| Yes | 10(76.9%) | 4(57.1%) |  |
| No | 3(23.1%) | 3(42.9%) |  |
| Tumor characteristics |  |  |  |
| AFP (ng/mL), n (%) |  |  | 0.658 |
| ≤400 ng/mL | 5(38.5%) | 2(28.6%) |  |
| >400ng/mL | 8(61.5%) | 5(71.4%) |  |
| Tumor size (cm), n (%) |  |  | 0.639 |
| ≤5cm | 3(23.1%) | 1(14.3%) |  |
| >5cm | 10(76.95%) | 6(85.7%) |  |
| Tumor number n (%) |  |  | 0.212 |
| Single | 12(92.3%) | 5(71.4%) |  |
| Multiple | 1(7.7%) | 2(28.6%) |  |
| child-pugh, n(%) |  |  | 0.452 |
| A | 12(92.3%) | 7(100%) |  |
| B | 1(7.7%) | 0 |  |
| MST(95% CI)(month) | 4.6(0.992-8.208) | 5.49(1.663-7.537) | 0.365 |

MST, median survival time, CI, confidence interval

TACE-Sor, transhepatic arterial chemoembolization combined with sorfenib.

**Supplement Table 2 Patient’s Characteristics and Survival for Type I PVTT patients received TACE combined with RT**

| Variables | TACE+Radiotheraphy |
| --- | --- |
|  | n=8 |
| Gendern , n(%) |  |
| Male | 7 |
| Female | 1 |
| Age, (years, n(%)) |  |
| ≤50 yrs, n (%) | 1 |
| >50 yrs, n (%) | 7 |
| Biochemistries |  |
| Total bilirubin(mmol/L), n(%) |  |
| ≦18.8 | 6 |
| > 18.8 | 2 |
| Albumin (g/l), n(%) |  |
| ≦34 | 0 |
| > 34 | 8 |
| ALT (u l/1), n(%) |  |
| ≦44 | 3 |
| > 44 | 5 |
| HbsAg n (%) |  |
| Positive | 7 |
| Negative | 1 |
| cirrhosis n (%) |  |
| Yes | 5 |
| No | 3 |
| Tumor characteristics |  |
| AFP (ng/mL), n (%) |  |
| ≤400 ng/mL | 6 |
| >400ng/mL | 2 |
| Tumor size (cm), n (%) |  |
| ≤5cm | 1 |
| >5cm | 7 |
| Tumor number n (%) |  |
| Single | 7 |
| Multiple | 1 |
| child-pugh, n(%) |  |
| A | 6 |
| B | 2 |
| MST(95% CI)(month) | 12.199(0-24.739) |

MST, median survival time, CI, confidence interval

**Supplement Table 3 Patient’s Characteristics for Surgery versus TACE**

|  | Before propensity matching | |  | After propensity matching | |  |
| --- | --- | --- | --- | --- | --- | --- |
| Variables | Surgery | TACE | P | Surgery | TACE | P |
| **Type I PVTT** | **(n=236)** | **(n=47)** |  | **(n=122 )** | **(n=45 )** | 0.920 |
| Gendern , n(%) |  |  | 0.344 |  |  |  |
| Male | 212(89.8%) | 40(85.1%) |  | 105(86.1%) | 39(86.7%) |  |
| Female | 24(10.2) | 7(14.9%) |  | 17(13.9%) | 6(13.3%) |  |
| Age, (years, n(%)) |  |  | **0.023** |  |  | 0.690 |
| ≤50 | 138(58.5%) | 19(40.4%) |  | 53(43.4%) | 18(40.0%) |  |
| >50 | 98(41.5%) | 28(59.6%) |  | 69(56.6%) | 27(60.0%) |  |
| Biochemistries |  |  |  |  |  |  |
| Total bilirubin(mmol/L), n(%) |  |  | 0.138 |  |  | 0.634 |
| ≤18.8 | 180(76.3%) | 31(66.0%) |  | 86(70.5%) | 30(66.7%) |  |
| > 18.8 | 56(23.7%) | 16(34.0%) |  | 36(29.5%) | 15(33.3%) |  |
| Albumin (g/l), n(%) |  |  | 0.119 |  |  | 0.446 |
| ≤34 | 8(3.4%) | 4(8.5%) |  | 5(4.1%) | 3(6.7%) |  |
| > 34 | 228(96.6%) | 43(91.5%) |  | 117(95.9%) | 42(93.3%) |  |
| ALT (u l/1), n(%) |  |  | 0.979 |  |  | 0.172 |
| ≤44 | 115(48.7%) | 23(48.9%) |  | 74(60.7%) | 22(48.9%) |  |
| > 44 | 121(51.3%) | 24(51.1%) |  | 48(39.3%) | 23(51.1%) |  |
| HbsAg n (%) |  |  | 0.295 |  |  | 0.896 |
| Positive | 213(90.3%) | 40(85.1%) |  | 102(83.6%) | 38(84.4%0 |  |
| Negative | 23(9.7%) | 7(14.9%) |  | 20(16.4%) | 7(15.6%) |  |
| cirrhosis n (%) |  |  | 0.839 |  |  | 0.655 |
| Yes | 152(64.4%) | 31(66.0%) |  | 74(60.7%) | 29(64.4%) |  |
| No | 84(35.6%) | 16(34.0%) |  | 48(39.3%) | 16(35.6%) |  |
| Tumor characteristics |  |  |  |  |  |  |
| AFP (ng/mL) , n (%) |  |  | 0.493 |  |  | 0.505 |
| ≤400 ng/mL | 83(35.2%) | 19(40.4%) |  | 42(34.4%) | 18(40.0%) |  |
| >400ng/mL | 153(64.8%) | 28(59.6%) |  | 80(65.6%) | 27(60.0%) |  |
| Tumor size (cm) , n (%) |  |  | 0.736 |  |  | 0.717 |
| ≤5cm | 50(21.2%) | 11(23.4%) |  | 24(19.7%) | 10(22.2%) |  |
| >5cm | 186(78.8%) | 36(76.6%) |  | 98(80.3%) | 35(77.8%) |  |
| Tumor number n (%) |  |  | 0.138 |  |  | 0.559 |
| Single | 217(91.9%) | 40(85.1%) |  | 112(91.8%) | 40(89.9%) |  |
| Multiple | 19(8.1%) | 7(14.9%) |  | 10(8.2%) | 5(11.1%) |  |
| child-pugh, n(%) |  |  | 1.000 |  |  | NA |
| A | 233(98.7%) | 47(100%) |  | 122(100%0 | 45(100%) |  |
| B | 3(1.3%) | 0 |  | 0 | 0 |  |
| **Type II PVTT** | **(n=315)** | **(n=288)** |  | **(n=187)** | **(n=187)** |  |
| Gendern , n(%) |  |  | 0.073 |  |  | 0.703 |
| Male | 294(93.3%) | 257(89.2%) |  | 173(92.5%) | 171(91.4%) |  |
| Female | 21(6.7%) | 31(10.8%) |  | 14(7.5%) | 16(8.6%) |  |
| Age, (years, n(%)) |  |  | **0.000** |  |  | 0.408 |
| ≤50 | 180(57.1%) | 123(42.7%) |  | 98(52.4%) | 90(48.1%) |  |
| >50 | 135(42.9%) | 165(57.3%) |  | 89(47.6%) | 97(51.9%) |  |
| Biochemistries |  |  |  |  |  |  |
| Total bilirubin(mmol/L), n(%) |  |  | **0.007** |  |  | 0.913 |
| ≤18.8 | 227(72.1%) | 178(61.8%) |  | 125(66.8%) | 124(66.3%) |  |
| > 18.8 | 88(27.9%) | 110(38.2%) |  | 62(33.2%) | 63(33.7%) |  |
| Albumin (g/l), n(%) |  |  | **0.000** |  |  | 0.748 |
| ≤34 | 22(7.0%) | 57(19.8%) |  | 21(11.2%) | 23(12.3%) |  |
| > 34 | 293(93.0%) | 231(80.2%) |  | 166(88.8%) | 164(87.7%) |  |
| ALT (u l/1), n(%) |  |  | 0.618 |  |  | 0.679 |
| ≤44 | 150(47.6%) | 143(49.7%) |  | 90(48.1%) | 94(50.3%) |  |
| > 44 | 165(52.4%) | 145(50.3%) |  | 97(51.9%) | 93(49.7%) |  |
| HbsAg n (%) |  |  | **0.009** |  |  | 0.748 |
| Positive | 286(90.8%) | 47(16.3%) |  | 166(88.8%) | 164(87.7%) |  |
| Negative | 29(9.2%) | 241(83.7%) |  | 21(11.2%) | 23(12.3%) |  |
| cirrhosis n (%) |  |  | **0.003** |  |  | 0.631 |
| Yes | 213(67.6%) | 226(78.5%) |  | 143(76.5%) | 139(74.3%) |  |
| No | 102(32.4%) | 62(21.5%) |  | 44(23.5%) | 48(25.7%) |  |
| Tumor characteristics |  |  |  |  |  |  |
| AFP (ng/mL) , n (%) |  |  | 0.237 |  |  | 0.751 |
| ≤400 | 110(34.9%) | 114(39.6%) |  | 72(38.5%) | 75(40.1%) |  |
| >400 | 205(65.1%) | 174(60.45) |  | 115(61.5%) | 112(59.9%) |  |
| Tumor size (cm) , n (%) |  |  | 0.076 |  |  | 0.882 |
| ≤5cm | 57(18.1%) | 37(12.8%) |  | 27(14.4%) | 26(13.9%) |  |
| >5cm | 258(81.9%) | 251(87.2%) |  | 160(85.6%) | 161(86.1%) |  |
| Tumor number n (%) |  |  | **0.000** |  |  | 0.748 |
| Single | 294(93.3%) | 192(66.7%) |  | 166(88.8%) | 164(87.7%) |  |
| Multiple | 21(6.7%) | 96(33.3%) |  | 21(11.2%) | 23(12.3%) |  |
| child-pugh, n(%) |  |  | **0.000** |  |  | 1.000 |
| A | 313(99.4%) | 272(94.4%) |  | 185(98.9%) | 184(98.45) |  |
| B | 2(0.6%) | 16(5.6%) |  | 2(1.1%) | 3(1.6%) |  |
| **Type III PVTT** | **(n=194)** | **(n=269)** |  | **(n=171)** | **(n=171)** |  |
| Gendern , n(%) |  |  | 0.721 |  |  | 0.868 |
| Male | 173(89.2%) | 237(88.1%) |  | 150(87.7%) | 151(88.3%) |  |
| Female | 21(10.8%) | 32(11.9%) |  | 21(12.3%) | 20(11.7%) |  |
| Age, (years, n(%)) |  |  | **0.037** |  |  | 0.317 |
| ≤50 | 122(62.9%) | 143(53.2%) |  | 110(64.3%) | 101(59.1%) |  |
| >50 | 72(37.1%) | 126(46.8%) |  | 61(35.7%) | 70(40.9%) |  |
| Biochemistries |  |  |  |  |  |  |
| Total bilirubin(mmol/L), n(%) |  |  | **0.000** |  |  | 0.564 |
| ≤18.8 | 136(70.1%) | 144(53.5%) |  | 113(66.1%) | 118(69.0%) |  |
| > 18.8 | 58(29.9%) | 125(46.5%) |  | 58(33.9%) | 53(31.0%) |  |
| Albumin (g/l), n(%) |  |  | **0.000** |  |  | 0.710 |
| ≤34 | 17(8.8%) | 69(25.7%) |  | 17(9.9%) | 15(8.8%) |  |
| > 34 | 177(91.2%) | 200(74.3%) |  | 154(90.1%) | 156(91.2%) |  |
| ALT (u l/1), n(%) |  |  | 0.060 |  |  | 0.742 |
| ≤44 | 83(42.8%) | 92(34.2(%) |  | 69(40.4%) | 72(42.1%) |  |
| > 44 | 111(57.2%) | 177(65.8%) |  | 102(59.6%) | 99(57.9%) |  |
| HbsAg n (%) |  |  | 0.476 |  |  | 0.540 |
| Positive | 171(88.1%) | 38(14.1%) |  | 148(86.5%) | 144(84.1%) |  |
| Negative | 23(11.9%) | 231(85.9%) |  | 23(13.5%) | 27(15.8%) |  |
| cirrhosis n (%) |  |  | 0.299 |  |  | 0.703 |
| Yes | 148(76.3%) | 216(80.3%) |  | 129(75.4%) | 132(77.2%) |  |
| No | 46(23.7%) | 53(19.7%) |  | 42(24.6%) | 39(22.8%) |  |
| Tumor characteristics |  |  |  |  |  |  |
| AFP (ng/mL) , n (%) |  |  | 0.214 |  |  | 0.741 |
| ≤400 | 81(41.8%) | 97(36.1%) |  | 71(41.5%) | 68(39.8%) |  |
| >400 | 113(58.2%) | 172(63.9%) |  | 100(58.5%) | 103(60.2%) |  |
| Tumor size (cm) , n (%) |  |  | 0.165 |  |  | 0.448 |
| ≤5cm | 31(16.0%) | 31(11.5%) |  | 28(16.4%) | 23(13.5%) |  |
| >5cm | 163(84.0%) | 238(88.5%) |  | 143(83.6%) | 148(86.5%) |  |
| Tumor number n (%) |  |  | 0.141 |  |  | 0.430 |
| Single | 182(93.8%) | 242(90.05) |  | 159(93.0%) | 155(90.6%) |  |
| Multiple | 12(6.2%) | 27(10.0%) |  | 12(7.0%) | 16(9.4%) |  |
| child-pugh, n(%) |  |  | 0.002 |  |  | 1.000 |
| A | 191(98.5%) | 248(92.2%) |  | 168(98.2%) | 167(97.7%) |  |
| B | 3(1.5%) | 21(7.8%) |  | 3(1.8%) | 4(2.3%) |  |

TACE, transhepatic arterial chemoembolization

**Supplement Table 4 Patient’s Characteristics for Surgery versus TACE+Sorafenib**

|  | Before propensity matching | |  | After propensity matching | |  |
| --- | --- | --- | --- | --- | --- | --- |
| Variables | Surgery | TACE+Sorafenib | P | Surgery | TACE+Sorafenib | P |
| **Type I PVTT** | **(n=236 )** | **(n=31 )** |  | **(n=51 )** | **(n=21 )** |  |
| Gendern , n(%) |  |  | 0.329 |  |  | 1.000 |
| Male | 212(89.8%) | 1(3.2%) |  | 47(94.0%) | 20(95.2%) |  |
| Female | 24(10.2) | 30(96.8%) |  | 3(6.0%) | 1(4.8%) |  |
| Age, (years, n(%)) |  |  | 0.159 |  |  | 0.446 |
| ≤50 | 138(58.5%) | 14(45.2%) |  | 29(58.0%) | 10(47.6%) |  |
| >50 | 98(41.5%) | 17(54.8%) |  | 21(42.0%) | 11(52.4%) |  |
| Biochemistries |  |  |  |  |  |  |
| Total bilirubin(mmol/L), n(%) |  |  | **0.000** |  |  | 0.290 |
| ≤18.8 | 180(76.3%) | 11(35.5%) |  | 32(64.0%) | 10(47.6%) |  |
| > 18.8 | 56(23.7%) | 20(64.5%) |  | 18(36.0%) | 11(52.4%) |  |
| Albumin (g/l), n(%) |  |  | **0.000** |  |  | 0.415 |
| ≤34 | 8(3.4%) | 24(77.4%) |  | 4(8.0%) | 3(14.3%) |  |
| > 34 | 228(96.6%) | 7(22.6%) |  | 46(92.0%) | 18(85.7%) |  |
| ALT (u l/1), n(%) |  |  | 0.079 |  |  | 0.800 |
| ≤44 | 115(48.7%) | 14(45.2%) |  | 28(56.0%) | 11(52.4%) |  |
| > 44 | 121(51.3%) | 17(54.8%) |  | 22(44.0%) | 10(47.65) |  |
| HbsAg n (%) |  |  | **0.000** |  |  | 1.000 |
| Positive | 213(90.3%) | 19(61.3%) |  | 16(32.0%) | 7(33.3%) |  |
| Negative | 23(9.7%) | 12(38.7%) |  | 34(68.0%) | 14(66.7%) |  |
| cirrhosis n (%) |  |  | 0.299 |  |  | 1.000 |
| Yes | 152(64.4%) | 17(54.8%) |  | 33(66.0%) | 14(66.75) |  |
| No | 84(35.6%) | 14(45.2%) |  | 17(34.0%) | 7(33.3%) |  |
| Tumor characteristics |  |  |  |  |  |  |
| AFP (ng/mL) , n (%) |  |  | 0.516 |  |  | 0.789 |
| ≤400 ng/mL | 83(35.2%) | 15(48.45) |  | 17(34.0%) | 8(38.1%) |  |
| >400ng/mL | 153(64.8%) | 16(51.6%) |  | 33(66.0%) | 13(61.9%) |  |
| Tumor size (cm) , n (%) |  |  | 0.859 |  |  | 0.574 |
| ≤5cm | 50(21.2%) | 7(22.6%) |  | 9(18.0%) | 5(23.8%) |  |
| >5cm | 186(78.8%) | 24(77.4%) |  | 41(82.0%) | 16(76.2%) |  |
| Tumor number n (%) |  |  | **0.000** |  |  | 1.000 |
| Single | 217(91.9%) | 16(51.6%) |  | 32(64.0%) | 14(66.7%) |  |
| Multiple | 19(8.1%) | 15(48.4%) |  | 18(36.0%) | 7(33.3%) |  |
| child-pugh, n(%) |  |  | 1.000 |  |  | NA |
| A | 233(98.7%) | 31(100%) |  | 50(100%) | 21(100%) |  |
| B | 3(1.3%) | 0 |  | 0 | 0 |  |
| **Type II PVTT** | **(n=315)** | **(n=45 )** |  | **(n=80)** | **(n=32 )** |  |
| Gendern , n(%) |  |  | 0.535 |  |  | 1.000 |
| Male | 294(93.3%) | 41(91.1%) |  | 75(93.8%) | 30(93.8%) |  |
| Female | 21(6.7%) | 4(8.9%) |  | 5(6.2%) | 2(6.2%) |  |
| Age, (years, n(%)) |  |  | 0.060 |  |  | 0.145 |
| ≤50 | 180(57.1%) | 19(42.2%) |  | 43(53.8%) | 12(37.5%) |  |
| >50 | 135(42.9%) | 26(57.8%) |  | 37(46.2%) | 20(62.5%) |  |
| Biochemistries |  |  |  |  |  |  |
| Total bilirubin(mmol/L), n(%) |  |  | **0.001** |  |  | 0.303 |
| ≤18.8 | 227(72.1%) | 21(46.7%) |  | 44(55.0%) | 14(43.8%) |  |
| > 18.8 | 88(27.9%) | 24(53.3%) |  | 36(45.0%) | 18(56.2%) |  |
| Albumin (g/l), n(%) |  |  | **0.048** |  |  | 1.000 |
| ≤34 | 22(7.0%) | 7(15.6%) |  | 10(12.5%) | 4(12.5%) |  |
| > 34 | 293(93.0%) | 38(84.4%) |  | 70(87.5%) | 28(87.5%) |  |
| ALT (u l/1), n(%) |  |  | 0.873 |  |  | 1.000 |
| ≤44 | 150(47.6%) | 22(48.9%) |  | 36(45.0%) | 14(43.8%) |  |
| > 44 | 165(52.4%) | 23(51.1%) |  | 44(55.0%) | 18(56.2%) |  |
| HbsAg n (%) |  |  | 0.184 |  |  | 0.774 |
| Positive | 286(90.8%) | 7(15.6%) |  | 67(83.8%) | 28(87.5%) |  |
| Negative | 29(9.2%) | 38(84.4%) |  | 13(16.2%) | 4(12.55) |  |
| cirrhosis n (%) |  |  | **0.014** |  |  | 1.000 |
| Yes | 213(67.6%) | 22(48.9%) |  | 44(55.0%) | 17(53.1%) |  |
| No | 102(32.4%) | 23(51.1%) |  | 36(45.0%) | 15(46.9%) |  |
| Tumor characteristics |  |  |  |  |  |  |
| AFP (ng/mL) , n (%) |  |  | 0.933 |  |  | 1.000 |
| ≤400 ng/mL | 110(34.9%) | 16(35.6%) |  | 32(40.0%) | 13(40.6%) |  |
| >400ng/mL | 205(65.1%) | 29(64.4%) |  | 48(60.0%) | 19(59.4%) |  |
| Tumor size (cm) , n (%) |  |  | **0.040** |  |  | 1.000 |
| ≤5cm | 57(18.1%) | 14(31.1%) |  | 22(27.5%) | 8(25.0%) |  |
| >5cm | 258(81.9%) | 31(68.9%) |  | 58(72.5%) | 24(75.0%) |  |
| Tumor number n (%) |  |  | **0.000** |  |  | 0.142 |
| Single | 294(93.3%) | 23(51.1%) |  | 64(80.0%) | 21(65.6%) |  |
| Multiple | 21(6.7%) | 22(48.9%) |  | 16(20.0%) | 11(34.4%) |  |
| child-pugh, n(%) |  |  | 0.078 |  |  | 1.000 |
| A | 313(99.4%) | 43(95.6%) |  | 78(97.5%) | 32(100%) |  |
| B | 2(0.6%) | 2(4.4%) |  | 2(2.5%) | 0 |  |
| **Type III PVTT** | **(n=194)** | **(n=37 )** |  | **(n=76)** | **(n=31 )** |  |
| Gendern , n(%) |  |  | 0.547 |  |  | 1.000 |
| Male | 173(89.2%) | 35(94.6%) |  | 70(92.1%) | 29(93.5%) |  |
| Female | 21(10.8%) | 2(5.4%) |  | 6(7.9%) | 2(6.5%) |  |
| Age, (years, n(%)) |  |  | 0.588 |  |  | 0.507 |
| ≤50 | 122(62.9%) | 25(67.6%) |  | 48(63.2%) | 22(71.0%) |  |
| >50 | 72(37.1%) | 12(32.4%) |  | 28(36.8%) | 9(29.0%) |  |
| Biochemistries |  |  |  |  |  |  |
| Total bilirubin(mmol/L), n(%) |  |  | **0.004** |  |  | 0.667 |
| ≤18.8 | 136(70.1%) | 17(45.9%) |  | 46(60.5%) | 17(54.8%) |  |
| > 18.8 | 58(29.9%) | 20(54.1%) |  | 30(39.5%) | 14(45.2%) |  |
| Albumin (g/l), n(%) |  |  | **0.021** |  |  | 0.776 |
| ≤34 | 17(8.8%) | 8(21.6%) |  | 12(15.8%) | 6(19.4%) |  |
| > 34 | 177(91.2%) | 29(78.4%) |  | 64(84.2%) | 25(80.6%) |  |
| ALT (u l/1), n(%) |  |  | 0.336 |  |  | 0.668 |
| ≤44 | 83(42.8%) | 19(51.4%) |  | 44(57.9%) | 16(51.6%) |  |
| > 44 | 111(57.2%) | 18(48.65) |  | 32(42.1%) | 15(48.4%) |  |
| HbsAg n (%) |  |  | 0.776 |  |  | 1.000 |
| Positive | 171(88.1%) | 3(8.1%) |  | 69(90.8%) | 28(90.3%) |  |
| Negative | 23(11.9%) | 34(91.9%) |  | 7(9.2%) | 3(9.7%) |  |
| cirrhosis n (%) |  |  | **0.005** |  |  | 0.510 |
| Yes | 148(76.3%) | 20(54.1%) |  | 50(65.8%) | 18(58.1%) |  |
| No | 46(23.7%) | 17(45.9%) |  | 26(34.2%) | 13(41.9%) |  |
| Tumor characteristics, n (%) |  |  |  |  |  |  |
| AFP (ng/mL) |  |  | 0.567 |  |  | 0.827 |
| ≤400 ng/mL | 81(41.8%) | 14(37.8%) |  | 30(39.5%) | 11(35.5%) |  |
| >400ng/mL | 113(58.2%) | 23(62.2%) |  | 46(60.5%) | 20(64.5%) |  |
| Tumor size (cm) , n (%) |  |  | 0.401 |  |  | 0.773 |
| ≤5cm | 31(16.0%) | 8(21.6%) |  | 13(17.1%) | 4(12.9%) |  |
| >5cm | 163(84.0%) | 29(78.4%) |  | 63(82.9%) | 27(87.1%) |  |
| Tumor number n (%) |  |  | **0.000** |  |  | 0.551 |
| Single | 182(93.8%) | 26(70.3%) |  | 67(88.2%) | 26(83.9%) |  |
| Multiple | 12(6.2%) | 11(29.7%) |  | 9(11.8%) | 5(16.1%) |  |
| child-pugh, n(%) |  |  | 0.505 |  |  | 1.000 |
| A | 191(98.5%) | 36(97.3%) |  | 74(97.4%) | 30(96.8%) |  |
| B | 3(1.5%) | 1(2.7%) |  | 2(2.6%) | 1(3.2%) |  |

TACE, transhepatic arterial chemoembolization

**Supplement Table 5 Patient’s Characteristics for Surgery versus TACE+Radiotheraphy**

|  | Before propensity matching | |  | After propensity matching | |  |
| --- | --- | --- | --- | --- | --- | --- |
| Variables | Surgery | TACE+RT | P | Surgery | TACE+RT | P |
| **Type II PVTT** | **(n=315 )** | **(n=54 )** |  | **(n=131)** | **(n=47 )** |  |
| Gendern , n(%) |  |  | 1.000 |  |  | 1.000 |
| Male | 294(93.3%) | 51(94.4%) |  | 125(95.4%) | 45(95.7%) |  |
| Female | 21(6.7%) | 3(5.6%) |  | 6(4.6%) | 2(4.3%) |  |
| Age, (years, n(%)) |  |  | **0.006** |  |  | 0.715 |
| ≤50 | 180(57.1%) | 20(37.0%) |  | 49(37.4%) | 19(40.4%) |  |
| >50 | 135(42.9%) | 34(63.0%) |  | 82(62.6%) | 28(59.6%) |  |
| Biochemistries |  |  |  |  |  |  |
| Total bilirubin(mmol/L), n(%) |  |  | 0.174 |  |  | 0.729 |
| ≤18.8 | 227(72.1%) | 34(63.0%) |  | 90(68.7%) | 31(66.0%) |  |
| > 18.8 | 88(27.9%) | 20(37.0%) |  | 41(31.3%) | 16(34.0%) |  |
| Albumin (g/l), n(%) |  |  | 1.000 |  |  | 0.383 |
| ≤34 | 22(7.0%) | 4(7.4%) |  | 4(3.1%) | 3(6.4%) |  |
| > 34 | 293(93.0%) | 50(92.6%) |  | 127(96.9%) | 44(93.6%) |  |
| ALT (u l/1), n(%) |  |  | 0.565 |  |  | 0.936 |
| ≤44 | 150(47.6%) | 28(51.9%) |  | 65(49.6%) | 23(48.9%) |  |
| > 44 | 165(52.4%) | 26(48.1%) |  | 66(50.4%) | 24(51.1%) |  |
| HbsAg n (%) |  |  | 0.659 |  |  | 1.000 |
| Positive | 286(90.8%) | 6(11.1%) |  | 118(90.1%) | 43(91.5%) |  |
| Negative | 29(9.2%) | 48(88.9%) |  | 13(9.9%) | 4(8.5%) |  |
| cirrhosis n (%) |  |  | 0.223 |  |  | 0.656 |
| Yes | 213(67.6%) | 41(75.9%) |  | 96(73.3%) | 36(76.6%) |  |
| No | 102(32.4%) | 13(24.1%) |  | 35(26.7%) | 11(23.4%) |  |
| Tumor characteristics |  |  |  |  |  |  |
| AFP (ng/mL) , n (%) |  |  | 0.109 |  |  | 0.457 |
| ≤400 ng/mL | 110(34.9%) | 25(46.3%) |  | 45(34.4%) | 19(40.4%) |  |
| >400ng/mL | 205(65.1%) | 29(53.7%) |  | 86(65.6%) | 28(59.6%) |  |
| Tumor size (cm) , n (%) |  |  | 0.690 |  |  | 0.521 |
| ≤5cm | 57(18.1%) | 11(20.4%) |  | 25(19.1%) | 7(14.9%) |  |
| >5cm | 258(81.9%) | 43(79.6%) |  | 106(80.9%) | 40(85.1%) |  |
| Tumor number n (%) |  |  | **0.004** |  |  | 0.275 |
| Single | 294(93.3%) | 44(81.55) |  | 119(90.8%) | 40(85.1%) |  |
| Multiple | 21(6.7%) | 10(18.5%) |  | 12(9.2%) | 7(14.9%) |  |
| child-pugh, n(%) |  |  | **0.024** |  |  | NA |
| A | 313(99.4%) | 51(94.4%) |  | 131(100%) | 47(100%) |  |
| B | 2(0.6%) | 3(5.6%) |  | 0 | 0 |  |
| **Type III PVTT** | **(n=194 )** | **(n=56 )** |  | **(n=50 )** | **(n=50 )** |  |
| Gendern , n(%) |  |  | 0.682 |  |  | 0.678 |
| Male | 173(89.2%) | 51(91.1%) |  | 48(96.0%) | 46(92.0%) |  |
| Female | 21(10.8%) | 5(8.9%) |  | 2(4.0%) | 4(8.0%) |  |
| Age, (years, n(%)) |  |  | 0.208 |  |  | 0.840 |
| ≤50 | 122(62.9%) | 30(53.6%) |  | 29(58.0%) | 28(56.0%) |  |
| >50 | 72(37.1%) | 26(46.4%) |  | 21(42.0%) | 22(44.0%) |  |
| Biochemistries |  |  |  |  |  |  |
| Total bilirubin(mmol/L), n(%) |  |  | 0.185 |  |  | 0.155 |
| ≤18.8 | 136(70.1%) | 34(60.7%) |  | 26(52.0%) | 33(66.0%) |  |
| > 18.8 | 58(29.9%) | 22(39.3%) |  | 24(48.0%) | 17(34.0%) |  |
| Albumin (g/l), n(%) |  |  | 1.000 |  |  | 0.495 |
| ≤34 | 17(8.8%) | 4(7.1%) |  | 0 | 2(4.0%) |  |
| > 34 | 177(91.2%) | 52(92.9%) |  | 50(100%) | 48(96.0%) |  |
| ALT (u l/1), n(%) |  |  | 0.992 |  |  | 0.410 |
| ≤44 | 83(42.8%) | 24(42.9%) |  | 17(34.0%) | 21(42.0%) |  |
| > 44 | 111(57.2%) | 32(57.1%) |  | 33(66.0%) | 29(58.0%) |  |
| HbsAg n (%) |  |  | 0.134 |  |  | 0.334 |
| Positive | 171(88.1%) | 11(19.6%) |  | 37(74.0%) | 41(82.0%) |  |
| Negative | 23(11.9%) | 45(80.4%) |  | 13(26.0%) | 9(18.0%) |  |
| cirrhosis n (%) |  |  | 0.637 |  |  | 0.822 |
| Yes | 148(76.3%) | 41(73.2%) |  | 37(74.0%) | 36(72.0%) |  |
| No | 46(23.7%) | 15(26.8%) |  | 13(26.0%) | 14(28.0%) |  |
| Tumor characteristics |  |  |  |  |  |  |
| AFP (ng/mL) , n (%) |  |  | 0.292 |  |  | 0.683 |
| ≤400 ng/mL | 81(41.8%) | 19(33.9%) |  | 21(42.0%) | 19(38.0%) |  |
| >400ng/mL | 113(58.2%) | 37(66.1%) |  | 29(58.0%) | 31(62.0%) |  |
| Tumor size (cm) , n (%) |  |  | 0.758 |  |  | 0.401 |
| ≤5cm | 31(16.0%) | 8(14.3%) |  | 9(18.0%) | 6(12.0%) |  |
| >5cm | 163(84.0%) | 48(85.75) |  | 41(82.0%) | 44(88.0%) |  |
| Tumor number n (%) |  |  | **0.019** |  |  | 0.318 |
| Single | 182(93.8%) | 47(83.9%) |  | 47(94.0%) | 43(86.0%) |  |
| Multiple | 12(6.2%) | 9(16.1%) |  | 3(6.0%) | 7(14.0%) |  |
| child-pugh, n(%) |  |  | **0.047** |  |  | NA |
| A | 191(98.5%) | 52(92.9%) |  | 50(100%) | 50(100%) |  |
| B | 3(1.5%) | 4(7.1%) |  | 0 | 0 |  |

RT, radiotherapy; TACE, transhepatic arterial chemoembolization

**Supplement Table 6 Patient’s Characteristics for TACE+Sorafenib versus TACE**

|  | Before propensity matching | |  | After propensity matching | |  |
| --- | --- | --- | --- | --- | --- | --- |
| Variables | TACE+Sorafenib | TACE | P | TACE+Sorafenib | TACE | P |
| **Type I PVTT** | **(n=31)** | **(n=47)** |  | **(n=15)** | **(n=15)** |  |
| Gendern , n(%) |  |  | 0.136 |  |  | 1.000 |
| Male | 1(3.2%) | 40(85.1%) |  | 13(86.7%) | 14(93.3%) |  |
| Female | 30(96.8%) | 7(14.9%) |  | 2(13.3%) | 1(6.7%) |  |
| Age, (years, n(%)) |  |  | 0.679 |  |  | 0.464 |
| ≤50 | 14(45.2%) | 19(40.4%) |  | 6(40.0%) | 8(53.3%) |  |
| >50 | 17(54.8%) | 28(59.6%) |  | 9(60.0%) | 7(46.7%) |  |
| Biochemistries |  |  |  |  |  |  |
| Total bilirubin(mmol/L), n(%) |  |  | **0.008** |  |  | 0.456 |
| ≤18.8 | 11(35.5%) | 31(66.0%) |  | 10(66.7%) | 8(53.3%) |  |
| > 18.8 | 20(64.5%) | 16(34.0%) |  | 5(33.3%) | 7(46.7%) |  |
| Albumin (g/l), n(%) |  |  | 0.103 |  |  | 1.000 |
| ≤34 | 24(77.4%) | 4(8.5%) |  | 3(20.0%) | 3(20.0%) |  |
| > 34 | 7(22.6%) | 43(91.5%) |  | 12(80.0%) | 12(80.0%) |  |
| ALT (u l/1), n(%) |  |  | 0.744 |  |  | 0.464 |
| ≤44 | 14(45.2%) | 23(48.9%) |  | 9(60.0%) | 7(46.7%) |  |
| > 44 | 17(54.8%) | 24(51.1%) |  | 6(40.0%) | 8(53.3%) |  |
| HbsAg n (%) |  |  | **0.016** |  |  | 0.682 |
| Positive | 19(61.3%) | 40(85.1%) |  | 10(66.7%) | 12(80.0%) |  |
| Negative | 12(38.7%) | 7(14.9%) |  | 5(33.3%) | 3(20.0%) |  |
| cirrhosis n (%) |  |  | 0.323 |  |  | 1.000 |
| Yes | 17(54.8%) | 31(66.0%) |  | 12(80.0%) | 11(73.3%) |  |
| No | 14(45.2%) | 16(34.0%) |  | 3(20.0%) | 4(26.7%) |  |
| Tumor characteristics |  |  |  |  |  |  |
| AFP (ng/mL) , n (%) |  |  | 0.488 |  |  | 0.705 |
| ≤400 ng/mL | 15(48.45) | 19(40.4%) |  | 5(33.3%) | 6(40.0%) |  |
| >400ng/mL | 16(51.6%) | 28(59.6%) |  | 10(66.7%) | 9(60.0%) |  |
| Tumor size (cm) , n (%) |  |  | 0.933 |  |  | 0.390 |
| ≤5cm | 7(22.6%) | 11(23.4%) |  | 2(13.3%) | 5(33.3%) |  |
| >5cm | 24(77.4%) | 36(76.6%) |  | 13(86.7%) | 10(66.7%) |  |
| Tumor number n (%) |  |  | **0.001** |  |  | 0.705 |
| Single | 16(51.6%) | 40(85.1%) |  | 10(66.7%) | 9(60.0%) |  |
| Multiple | 15(48.4%) | 7(14.9%) |  | 5(33.3%) | 6(40.0%) |  |
| child-pugh, n(%) |  |  |  |  |  | NA |
| A | 31(100%) | 47(100%) | NA | 15(100%) | 15(100%) |  |
| B | 0 | 0 |  | 0 | 0 |  |
| **Type II PVTT** | **(n=45)** | **(n=288)** |  | **(n=36)** | **(n=99)** |  |
| Gendern , n(%) |  |  | 1.000 |  |  | 1.000 |
| Male | 41(91.1%) | 257(89.2%) |  | 32(88.9%) | 89(89.9%) |  |
| Female | 4(8.9%) | 31(10.8%) |  | 4(11.1%) | 10(10.1%) |  |
| Age, (years, n(%)) |  |  | 0.951 |  |  | 0.659 |
| ≤50 | 19(42.2%) | 123(42.7%) |  | 17(47.2%) | 51(51.5%) |  |
| >50 | 26(57.8%) | 165(57.3%) |  | 19(52.8%) | 48(48.5%) |  |
| Biochemistries |  |  |  |  |  |  |
| Total bilirubin(mmol/L), n(%) |  |  | 0.054 |  |  | 0.253 |
| ≤18.8 | 21(46.7%) | 178(61.8%) |  | 16(44.4%) | 55(55.6%) |  |
| > 18.8 | 24(53.3%) | 110(38.2%) |  | 20(55.6%) | 44(44.4%) |  |
| Albumin (g/l), n(%) |  |  | 0.502 |  |  | 0.738 |
| ≤34 | 7(15.6%) | 57(19.8%) |  | 6(16.7%) | 19(19.2%) |  |
| > 34 | 38(84.4%) | 231(80.2%) |  | 30(83.3%) | 80(80.8%) |  |
| ALT (u l/1), n(%) |  |  | 0.924 |  |  | 0.433 |
| ≤44 | 22(48.9%) | 143(49.7%) |  | 18(50.0%) | 57(57.6%) |  |
| > 44 | 23(51.1%) | 145(50.3%) |  | 18(50.0%) | 42(42.4%) |  |
| HbsAg n (%) |  |  | 0.897 |  |  | 0.974 |
| Positive | 7(15.6%) | 47(16.3%) |  | 29(80.6%) | 80(80.8%) |  |
| Negative | 38(84.4%) | 241(83.7%) |  | 7(19.4%) | 19(19.2%) |  |
| cirrhosis n (%) |  |  | **0.000** |  |  | 0.958 |
| Yes | 22(48.9%) | 226(78.5%) |  | 22(61.1%) | 60(60.6%) |  |
| No | 23(51.1%) | 62(21.5%) |  | 14(38.9%) | 39(39.4%) |  |
| Tumor characteristics |  |  |  |  |  |  |
| AFP (ng/mL) , n (%) |  |  | 0.607 |  |  | 0.849 |
| ≤400 ng/mL | 16(35.6%) | 114(39.6%) |  | 13(36.1%) | 34(34.3%) |  |
| >400ng/mL | 29(64.4%) | 174(60.45) |  | 23(63.9%) | 65(65.7%) |  |
| Tumor size (cm) , n (%) |  |  | **0.002** |  |  | 0.715 |
| ≤5cm | 14(31.1%) | 37(12.8%) |  | 6(16.7%) | 14(14.1%) |  |
| >5cm | 31(68.9%) | 251(87.2%) |  | 30(83.3%) | 85(85.9%) |  |
| Tumor number n (%) |  |  | **0.042** |  |  | 0.650 |
| Single | 23(51.1%) | 192(66.7%) |  | 21(58.3%) | 62(62.6%) |  |
| Multiple | 22(48.9%) | 96(33.3%) |  | 15(41.7%) | 37(37.4%) |  |
| child-pugh, n(%) |  |  | 1.000 |  |  | 1.000 |
| A | 43(95.6%) | 272(94.4%) |  | 35(97.2%) | 95(96.0%) |  |
| B | 2(4.4%) | 16(5.6%) |  | 1(2.8%) | 4(4.0%) |  |
| **Type III PVTT** | **(n=37)** | **(n=269)** |  | **(n=32)** | **(n=87)** |  |
| Gendern , n(%) |  |  | 0.400 |  |  | 1.000 |
| Male | 35(94.6%) | 237(88.1%) |  | 30(93.8%) | 81(93.1%) |  |
| Female | 2(5.4%) | 32(11.9%) |  | 2(6.2%) | 6(6.9%) |  |
| Age, (years, n(%)) |  |  | 0.099 |  |  | 0.576 |
| ≤50 | 25(67.6%) | 143(53.2%) |  | 22(68.8%) | 55(63.2%) |  |
| >50 | 12(32.4%) | 126(46.8%) |  | 10(31.2%) | 32(36.8%) |  |
| Biochemistries |  |  |  |  |  |  |
| Total bilirubin(mmol/L), n(%) |  |  | 0.386 |  |  | 0.253 |
| ≤18.8 | 17(45.9%) | 144(53.5%) |  | 17(53.2%) | 36(41.4%) |  |
| > 18.8 | 20(54.1%) | 125(46.5%) |  | 15(46.9%) | 51(58.6%) |  |
| Albumin (g/l), n(%) |  |  | 0.596 |  |  | 0.670 |
| ≤34 | 8(21.6%) | 69(25.7%) |  | 7(21.9%) | 16(18.4%) |  |
| > 34 | 29(78.4%) | 200(74.3%) |  | 25(78.1%) | 71(81.6%) |  |
| ALT (u l/1), n(%) |  |  | **0.042** |  |  | 0.253 |
| ≤44 | 19(51.4%) | 92(34.2(%) |  | 17(53.1%) | 36(41.4%) |  |
| > 44 | 18(48.65) | 177(65.8%) |  | 15(46.9%) | 51(58.6%) |  |
| HbsAg n (%) |  |  | 0.442 |  |  | 0.757 |
| Positive | 3(8.1%) | 38(14.1%) |  | 29(90.6%) | 76(87.4%) |  |
| Negative | 34(91.9%) | 231(85.9%) |  | 3(9.4%) | 11(12.6%) |  |
| cirrhosis n (%) |  |  | **0.000** |  |  | 0.943 |
| Yes | 20(54.1%) | 216(80.3%) |  | 20(62.5%) | 55(63.2%) |  |
| No | 17(45.9%) | 53(19.7%) |  | 12(37.5%) | 32(36.8%) |  |
| Tumor characteristics |  |  |  |  |  |  |
| AFP (ng/mL) , n (%) |  |  | 0.833 |  |  | 0.991 |
| ≤400 ng/mL | 14(37.8%) | 97(36.1%) |  | 11(34.4%) | 30(34.5%) |  |
| >400ng/mL | 23(62.2%) | 172(63.9%) |  | 21(65.6%) | 57(65.5%) |  |
| Tumor size (cm) , n (%) |  |  | 0.084 |  |  | 0.731 |
| ≤5cm | 8(21.6%) | 31(11.5%) |  | 6(18.8%) | 14(16.1%) |  |
| >5cm | 29(78.4%) | 238(88.5%) |  | 26(81.2%) | 73(83.9%) |  |
| Tumor number n (%) |  |  | **0.001** |  |  | 0.997 |
| Single | 26(70.3%) | 242(90.05) |  | 25(78.1%) | 68(78.2%) |  |
| Multiple | 11(29.7%) | 27(10.0%) |  | 7(21.9%) | 19(21.8%) |  |
| child-pugh, n(%) |  |  | 0.493 |  |  | 1.000 |
| A | 36(97.3%) | 248(92.2%) |  | 31(96.9%) | 82(94.3%) |  |
| B | 1(2.7%) | 21(7.8%) |  | 1(3.1%) | 5(5.7%) |  |

TACE, transhepatic arterial chemoembolization

**Supplement Table 7 Patient’s Characteristics for TACE+Sorafenib versus TACE+Radiotheraphy**

|  | Before propensity matching | |  | After propensity matching | |  |
| --- | --- | --- | --- | --- | --- | --- |
| Variables | TACE+Sorafenib | TACE+RT | P | TACE+Sorafenib | TACE+RT | P |
| **Type II PVTT** | **(n=45 )** | **(n=54 )** |  | **(n=23 )** | **(n=23 )** |  |
| Gendern , n(%) |  |  | 0.699 |  |  | 1.000 |
| Male | 41(91.1%) | 51(94.4%) |  | 20(87.0%) | 20(87.0%) |  |
| Female | 4(8.9%) | 3(5.6%) |  | 3(13.0%) | 3(13.0%) |  |
| Age, (years, n(%)) |  |  | 0.599 |  |  | 0.234 |
| ≤50 | 19(42.2%) | 20(37.0%) |  | 12(52.2%) | 8(34.8%) |  |
| >50 | 26(57.8%) | 34(63.0%) |  | 11(47.8%) | 15(65.2%) |  |
| Biochemistries |  |  |  |  |  |  |
| Total bilirubin(mmol/L), n(%) |  |  | 0.104 |  |  | 0.768 |
| ≤18.8 | 21(46.7%) | 34(63.0%) |  | 11(47.8%) | 12(52.2%) |  |
| > 18.8 | 24(53.3%) | 20(37.0%) |  | 12(52.2%) | 11(47.8%) |  |
| Albumin (g/l), n(%) |  |  | 0.219 |  |  | 1.000 |
| ≤34 | 7(15.6%) | 4(7.4%) |  | 2(8.7%) | 1(4.3%) |  |
| > 34 | 38(84.4%) | 50(92.6%) |  | 21(91.3%) | 22(95.7%) |  |
| ALT (u l/1), n(%) |  |  | 0.769 |  |  | 0.768 |
| ≤44 | 22(48.9%) | 28(51.9%) |  | 11(47.8%) | 12(52.2%) |  |
| > 44 | 23(51.1%) | 26(48.1%) |  | 12(52.2%) | 11(47.8%) |  |
| HbsAg n (%) |  |  | 0.514 |  |  | 0.665 |
| Positive | 7(15.6%) | 6(11.1%) |  | 19(2.6%) | 21(91.3%) |  |
| Negative | 38(84.4%) | 48(88.9%) |  | 4(17.4%) | 2(8.7%) |  |
| cirrhosis n (%) |  |  | **0.005** |  |  | 0.369 |
| Yes | 22(48.9%) | 41(75.9%) |  | 15(65.2%) | 12(52.2%) |  |
| No | 23(51.1%) | 13(24.1%) |  | 8(34.8%) | 11(47.8%) |  |
| Tumor characteristics |  |  |  |  |  |  |
| AFP (ng/mL) , n (%) |  |  | 0.280 |  |  | 0.543 |
| ≤400 ng/mL | 16(35.6%) | 25(46.3%) |  | 8(34.8%) | 10(43.5%) |  |
| >400ng/mL | 29(64.4%) | 29(53.7%) |  | 15(65.2%) | 13(56.5%) |  |
| Tumor size (cm) , n (%) |  |  | 0.221 |  |  | 1.000 |
| ≤5cm | 14(31.1%) | 11(20.4%) |  | 5(21.7%) | 4(17.4%) |  |
| >5cm | 31(68.9%) | 43(79.6%) |  | 18(78.3%) | 19(82.6%) |  |
| Tumor number n (%) |  |  | **0.001** |  |  | 0.502 |
| Single | 23(51.1%) | 44(81.55) |  | 18(78.3%) | 16(69.6%) |  |
| Multiple | 22(48.9%) | 10(18.5%) |  | 5(21.7%) | 7(30.4%) |  |
| child-pugh, n(%) |  |  | 1.000 |  |  | NA |
| A | 43(95.6%) | 51(94.4%) |  | 23(100%) | 23(100%) |  |
| B | 2(4.4%) | 3(5.6%) |  | 0 | 0 |  |
| **Type III PVTT** | **(n=37)** | **(n=56 )** |  |  |  |  |
| Gendern , n(%) |  |  | 0.699 |  |  |  |
| Male | 35(94.6%) | 51(91.1%) |  |  |  |  |
| Female | 2(5.4%) | 5(8.9%) |  |  |  |  |
| Age, (years, n(%)) |  |  | 0.179 |  |  |  |
| ≤50 | 25(67.6%) | 30(53.6%) |  |  |  |  |
| >50 | 12(32.4%) | 26(46.4%) |  |  |  |  |
| Biochemistries |  |  |  |  |  |  |
| Total bilirubin(mmol/L), n(%) |  |  | 0.161 |  |  |  |
| ≤18.8 | 17(45.9%) | 34(60.7%) |  |  |  |  |
| > 18.8 | 20(54.1%) | 22(39.3%) |  |  |  |  |
| Albumin (g/l), n(%) |  |  | 0.058 |  |  |  |
| ≤34 | 8(21.6%) | 4(7.1%) |  |  |  |  |
| > 34 | 29(78.4%) | 52(92.9%) |  |  |  |  |
| ALT (u l/1), n(%) |  |  | 0.421 |  |  |  |
| ≤44 | 19(51.4%) | 24(42.9%) |  |  |  |  |
| > 44 | 18(48.65) | 32(57.1%) |  |  |  |  |
| HbsAg n (%) |  |  | 0.151 |  |  |  |
| Positive | 3(8.1%) | 11(19.6%) |  |  |  |  |
| Negative | 34(91.9%) | 45(80.4%) |  |  |  |  |
| cirrhosis n (%) |  |  | 0.057 |  |  |  |
| Yes | 20(54.1%) | 41(73.2%) |  |  |  |  |
| No | 17(45.9%) | 15(26.8%) |  |  |  |  |
| Tumor characteristics |  |  |  |  |  |  |
| AFP (ng/mL) , n (%) |  |  | 0.700 |  |  |  |
| ≤400 ng/mL | 14(37.8%) | 19(33.9%) |  |  |  |  |
| >400ng/mL | 23(62.2%) | 37(66.1%) |  |  |  |  |
| Tumor size (cm) , n (%) |  |  | 0.359 |  |  |  |
| ≤5cm | 8(21.6%) | 8(14.3%) |  |  |  |  |
| >5cm | 29(78.4%) | 48(85.75) |  |  |  |  |
| Tumor number n (%) |  |  | 0.117 |  |  |  |
| Single | 26(70.3%) | 47(83.9%) |  |  |  |  |
| Multiple | 11(29.7%) | 9(16.1%) |  |  |  |  |
| child-pugh, n(%) |  |  | 0.645 |  |  |  |
| A | 36(97.3%) | 52(92.9%) |  |  |  |  |
| B | 1(2.7%) | 4(7.1%) |  |  |  |  |

RT, radiotherapy; TACE, transhepatic arterial chemoembolization

**Supplement Table 8 Patient’s Characteristics for TACE+Radiotheraphy versus TACE**

|  | Before propensity matching | |  | After propensity matching | |  |
| --- | --- | --- | --- | --- | --- | --- |
| Variables | TACE+Radiotheraphy | TACE | P | TACE+Radiotheraphy | TACE | P |
| **Type II PVTT** | **(n=54 )** | **(n=288 )** |  | **(n=52 )** | **(n=144 )** |  |
| Gendern , n(%) |  |  | 0.324 |  |  | 0.702 |
| Male | 51(94.4%) | 257(89.2%) |  | 49(94.2%) | 138(95.8%) |  |
| Female | 3(5.6%) | 31(10.8%) |  | 3(5.8%) | 6(4.2%) |  |
| Age, (years, n(%)) |  |  | 0.438 |  |  | 0.752 |
| ≤50 | 20(37.0%) | 123(42.7%) |  | 20(38.5%) | 59(41.0%) |  |
| >50 | 34(63.0%) | 165(57.3%) |  | 32(61.5%) | 85(59.0%) |  |
| Biochemistries |  |  |  |  |  |  |
| Total bilirubin(mmol/L), n(%) |  |  | 0.872 |  |  | 0.917 |
| ≤18.8 | 34(63.0%) | 178(61.8%) |  | 34(65.4%) | 93(64.6%) |  |
| > 18.8 | 20(37.0%) | 110(38.2%) |  | 18(34.6%) | 51(35.4%) |  |
| Albumin (g/l), n(%) |  |  | **0.032** |  |  | 0.600 |
| ≤34 | 4(7.4%) | 57(19.8%) |  | 4(7.7%) | 16(11.1%) |  |
| > 34 | 50(92.6%) | 231(80.2%) |  | 48(92.3%) | 128(88.9%) |  |
| ALT (u l/1), n(%) |  |  | 0.768 |  |  | 0.365 |
| ≤44 | 28(51.9%) | 143(49.7%) |  | 28(53.8%) | 67(46.5%) |  |
| > 44 | 26(48.1%) | 145(50.3%) |  | 24(46.2%) | 77(53.5%) |  |
| HbsAg n (%) |  |  | 0.332 |  |  | 0.778 |
| Positive | 6(11.1%) | 47(16.3%) |  | 47(90.4%) | 132(91.7%) |  |
| Negative | 48(88.9%) | 241(83.7%) |  | 5(9.6%) | 12(8.3%) |  |
| cirrhosis n (%) |  |  | 0.678 |  |  | 0.735 |
| Yes | 41(75.9%) | 226(78.5%) |  | 40(76.9%) | 114(79.2%) |  |
| No | 13(24.1%) | 62(21.5%) |  | 12(23.1%) | 30(20.8%) |  |
| Tumor characteristics |  |  |  |  |  |  |
| AFP (ng/mL) , n (%) |  |  | 0.357 |  |  | 0.968 |
| ≤400 ng/mL | 25(46.3%) | 114(39.6%) |  | 24(46.2%) | 66(45.8%) |  |
| >400ng/mL | 29(53.7%) | 174(60.45) |  | 28(53.8%) | 78(54.2%) |  |
| Tumor size (cm) , n (%) |  |  | 0.144 |  |  | 0.818 |
| ≤5cm | 11(20.4%) | 37(12.8%) |  | 9(17.3%) | 27(18.8%) |  |
| >5cm | 43(79.6%) | 251(87.2%) |  | 43(82.7%) | 117(81.2%) |  |
| Tumor number n (%) |  |  | **0.031** |  |  | 0.582 |
| Single | 44(81.55) | 192(66.7%) |  | 42(80.8%) | 111(77.1%) |  |
| Multiple | 10(18.5%) | 96(33.3%) |  | 10(19.2%) | 33(22.9%) |  |
| child-pugh, n(%) |  |  | 1.000 |  |  | 1.000 |
| A | 51(94.4%) | 272(94.4%) |  | 50(96.2%) | 136(94.4%) |  |
| B | 3(5.6%) | 16(5.6%) |  | 2(3.8%) | 8(5.6%) |  |
| **Type III PVTT** | **(n=56)** | **(n=269)** |  | **(n=54 )** | **(n=148 )** |  |
| Gendern , n(%) |  |  | 0.525 |  |  | 0.916 |
| Male | 51(91.1%) | 237(88.1%) |  | 49(90.7%) | 135(91.2%) |  |
| Female | 5(8.9%) | 32(11.9%) |  | 5(9.3%) | 13(8.8%) |  |
| Age, (years, n(%)) |  |  | 0.955 |  |  | 0.985 |
| ≤50 | 30(53.6%) | 143(53.2%) |  | 30(55.6%) | 82(55.4%) |  |
| >50 | 26(46.4%) | 126(46.8%) |  | 24(44.4%) | 66(44.6%) |  |
| Biochemistries |  |  |  |  |  |  |
| Total bilirubin(mmol/L), n(%) |  |  | 0.326 |  |  | 0.643 |
| ≤18.8 | 34(60.7%) | 144(53.5%) |  | 32(59.3%) | 93(62.8%) |  |
| > 18.8 | 22(39.3%) | 125(46.5%) |  | 22(40.7%) | 55(37.2%) |  |
| Albumin (g/l), n(%) |  |  | **0.001** |  |  | 1.000 |
| ≤34 | 4(7.1%) | 69(25.7%) |  | 4(7.4%) | 10(6.8%) |  |
| > 34 | 52(92.9%) | 200(74.3%) |  | 50(92.6%) | 138(93.2%) |  |
| ALT (u l/1), n(%) |  |  | 0.219 |  |  | 0.521 |
| ≤44 | 24(42.9%) | 92(34.2(%) |  | 22(40.7%) | 53(35.8%) |  |
| > 44 | 32(57.1%) | 177(65.8%) |  | 32(59.3%) | 95(64.2%) |  |
| HbsAg n (%) |  |  | 0.294 |  |  | 0.787 |
| Positive | 11(19.6%) | 38(14.1%) |  | 44(81.5%) | 123(83.1%) |  |
| Negative | 45(80.4%) | 231(85.9%) |  | 10(18.5%) | 25(16.9%) |  |
| cirrhosis n (%) |  |  | 0.236 |  |  | 0.870 |
| Yes | 41(73.2%) | 216(80.3%) |  | 41(75.9%) | 114(77.0%) |  |
| No | 15(26.8%) | 53(19.7%) |  | 13(24.1%) | 34(23.0%) |  |
| Tumor characteristics |  |  |  |  |  |  |
| AFP (ng/mL) , n (%) |  |  | 0.762 |  |  | 0.952 |
| ≤400 ng/mL | 19(33.9%) | 97(36.1%) |  | 18(33.3%) | 50(33.8%) |  |
| >400ng/mL | 37(66.1%) | 172(63.9%) |  | 36(66.7%) | 98(66.2%) |  |
| Tumor size (cm) , n (%) |  |  | 0.563 |  |  | 0.878 |
| ≤5cm | 8(14.3%) | 31(11.5%) |  | 7(13.0%) | 18(12.2%) |  |
| >5cm | 48(85.75) | 238(88.5%) |  | 47(87.0%) | 130(87.8%) |  |
| Tumor number n (%) |  |  | 0.191 |  |  | 0.436 |
| Single | 47(83.9%) | 242(90.05) |  | 46(85.2%) | 132(89.2%) |  |
| Multiple | 9(16.1%) | 27(10.0%) |  | 8(14.8%) | 16(10.8%) |  |
| child-pugh, n(%) |  |  | 1.000 |  |  | 0.462 |
| A | 52(92.9%) | 248(92.2%) |  | 50(92.6%) | 142(95.9%) |  |
| B | 4(7.1%) | 21(7.8%) |  | 4(7.4%) | 6(4.1%) |  |

RT, radiotherapy; TACE, transhepatic arterial chemoembolization
